# Heteroleptic actinocenes: a thorium(iv)–cyclobutadienyl–cyclooctatetraenyl–di-potassium-cyclooctatetraenyl complex†

**DOI:** 10.1039/d0sc02479a

**Published:** 2020-06-10

**Authors:** Josef T. Boronski, Ashley J. Wooles, Stephen T. Liddle

**Affiliations:** Department of Chemistry, The University of Manchester Oxford Road Manchester M13 9PL UK steve.liddle@manchester.ac.uk

## Abstract

Despite the vast array of η^*n*^-carbocyclic C_5–8_ complexes reported for actinides, cyclobutadienyl (C_4_) remain exceedingly rare, being restricted to six uranium examples. Here, overcoming the inherent challenges of installing highly reducing C_4_-ligands onto actinides when using polar starting materials such as halides, we report that reaction of [Th(η^8^-C_8_H_8_)_2_] with [K_2_{C_4_(SiMe_3_)_4_}] gives [{Th(η^4^-C_4_[SiMe_3_]_4_)(μ-η^8^-C_8_H_8_)(μ-η^2^-C_8_H_8_)(K[C_6_H_5_Me]_2_)}_2_{K(C_6_H_5_Me)}{K}] (**1**), a new type of heteroleptic actinocene. Quantum chemical calculations suggest that the thorium ion engages in π- and δ-bonding to the η^4^-cyclobutadienyl and η^8^-cyclooctatetraenyl ligands, respectively. Furthermore, the coordination sphere of this bent thorocene analogue is supplemented by an η^2^-cyclooctatetraenyl interaction, which calculations suggest is composed of σ- and π-symmetry donations from in-plane in- and out-of-phase C=C 2p-orbital combinations to vacant thorium 6d orbitals. The characterisation data are consistent with this being a metal–alkene-type interaction that is integral to the bent structure and stability of this complex.

## Abstract 2

We report the first thorium–cyclobutadienyl complex, a new type of heteroleptic actinocene, that exhibits ‘an alkene-like’ thorium–η^2^-cyclooctatetraenyl interaction.

## Graphical abstract

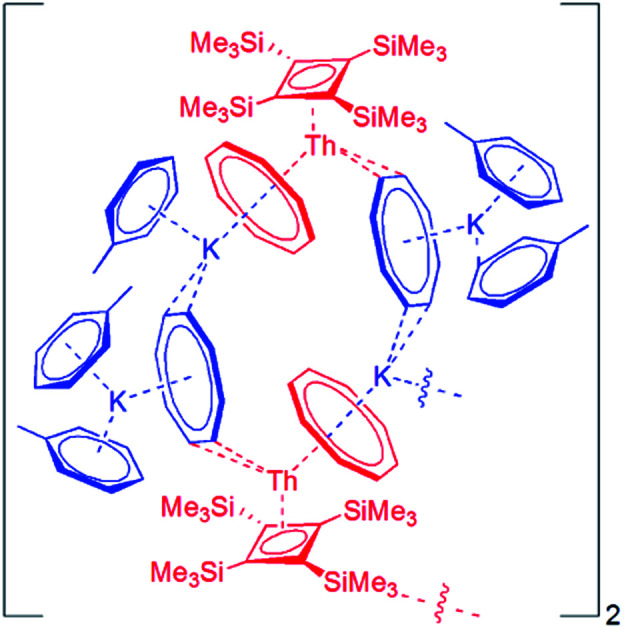

## Introduction

The synthesis of uranocene, [U(η^8^-C_8_H_8_)_2_], the first actinocene, was reported in 1968, and its cousin thorocene, [Th(η^8^-C_8_H_8_)_2_], emerged in 1969.^1^ Uranocene was a landmark discovery, not only in the field of f-element chemistry, but organometallic chemistry as a whole, and it inspired research into the degree of 5f/6d orbital participation in metal–ligand bonding that is still burgeoning today.^2^ Furthermore, due to the steric and electronic versatility of cyclooctatetraenyl dianions, which provide four-fold symmetry bonding combinations with 5f/6d orbitals, this ligand class has been used widely in organoactinide chemistry,^2*d*,3^ with uranium- and thorium–cyclooctatetraenyl complexes being found to exhibit uncommon bonding, oxidation state, and ligand motifs.^4^

Despite advances in actinide science enabled by cyclooctatetraenyl ligands, the closely-related, but far smaller, dianionic cyclobutadienyl ligand, which also provides up to four-fold symmetry bonding combinations to metals, has, thus far, remained barely investigated. This possibly reflects the paucity of suitable starting materials and the proclivity of cyclobutadienyls to decompose *via* reductive, protonolysis, or cyclometallation routes when in the coordination sphere of polar f-elements. Indeed, although transition metal cyclobutadienyl chemistry was established in the 1960s,^5^ the first f-element-cyclobutadienyl complex, an inverted sandwich tetraphenylcyclobutadienyl diuranium(iv) species, was reported in 2013.^6,7^ Very recently, the uranium(iv)–cyclobutadienyl half sandwich pianostool complex [U{C_4_(SiMe_3_)_4_}(BH_4_)_3_][Li(THF)_4_] (**A**) was reported,^8^ and shortly after that four uranium(iv)–cyclobutadienyl complexes [U{C_4_(SiMe_3_)_4_}(BH_4_)_3_][Na(12-crown-4)],^9^ [U{C_4_(SiMe_3_)_4_}(BH_4_)_2_(μ-BH_4_){K(THF)_2_}]_2_,^9^ [U(BH_4_){C_4_(SiMe_3_)_4_}{κ^3^-C_4_H(SiMe_3_)_3_-κ-(CH_2_SiMe_2_)}][Na(^*t*^BuOMe)_3.6_(THF)_0.4_],^9^ and [{U(C_4_[SiMe_3_]_4_)(μ-I)_2_}_3_{μ_3_-O}][Mg(THF)_6_] were disclosed.^10^ These six uranium–cyclobutadienyl complexes constitute all actinide–cyclobutadienyl chemistry to date, in stark contrast to the plethora of reported f-element η^*n*^-carbocyclic C_5–9_ complexes.^11^ Thus, a thorium–cyclobutadienyl complex of any kind is conspicuous by its absence, but realising such a target would provide comparisons between uranium and thorium and provide further insight into actinide–cyclobutadienyl bonding.

Herein, we report the synthesis of the first thorium–cyclobutadienyl complex, which, containing cyclobutadienyl and cyclooctatetraenyl ligands, is an unprecedented heteroleptic actinocene analogue. The thorium ion engages in π- and δ-bonding to the cyclobutadienyl and cyclooctatetraenyl ligands, respectively, and the coordination sphere of this bent thorocene analogue is supplemented by an η^2^-cyclooctatetraenyl interaction from co-complexed dipotassium-cyclooctatetraenyl. Quantum chemical studies reveal that this thorium–η^2^-cyclooctatetraenyl interaction is composed of σ- and π-symmetry alkene-like donation from in-plane in- and out-of-phase C=C 2p-orbital combinations to formally vacant thorium 6d orbitals.

## Results and discussion

### Synthetic considerations

We previously reported that reaction of [Li_2_{C_4_(SiMe_3_)_4_}(THF)_2_]^12^ with UCl_4_ led to reduction of uranium(iv) to intractable mixtures of uranium(iii)-containing products.^8^ However, thorium(iv) has a far greater reduction potential (*E*^θ^ Th(iv) → Th(iii) −3.7 V) than that of uranium(iv) (*E*^θ^ U(iv) → U(iii) −0.6 V).^13^ Nevertheless, addition of a solution of [K_2_{C_4_(SiMe_3_)_4_}]^14^ to [ThCl_4_(THF)_3.5_]^15^ resulted in oxidation of dianionic {C_4_(SiMe_3_)_4_}^2−^ to the neutral cyclobutadiene {C_4_(SiMe_3_)_4_},^16^ as confirmed by multinuclear NMR spectroscopy, and deposition of an insoluble dark grey precipitate presumed to be KCl and colloidal thorium.^17^ This underscores the strongly reducing nature of {C_4_(SiMe_3_)_4_}^2−^ and led us to conclude that polar actinide halides are unsuitable starting materials for our purposes. Advancing an alternate strategy, it was reasoned that less polar thorium(iv)–carbocyclic complexes would be less prone to the undesirable redox chemistry observed for actinide-halides. On hard-soft acid-base theory grounds, [Th(η^8^-C_8_H_8_)_2_] was selected as a starting material^1,18^ since we anticipated that displacement of the soft, charge-diffuse 10π-{C_8_H_8_}^2−^ dianion (1.25e^−^ per carbon) by the hard, charge-concentrated 6π-{C_4_(SiMe_3_)_4_}^2−^ dianion (1.5e^−^ per carbon) would be favourable for the hard thorium(iv) ion.

**Scheme 1:**
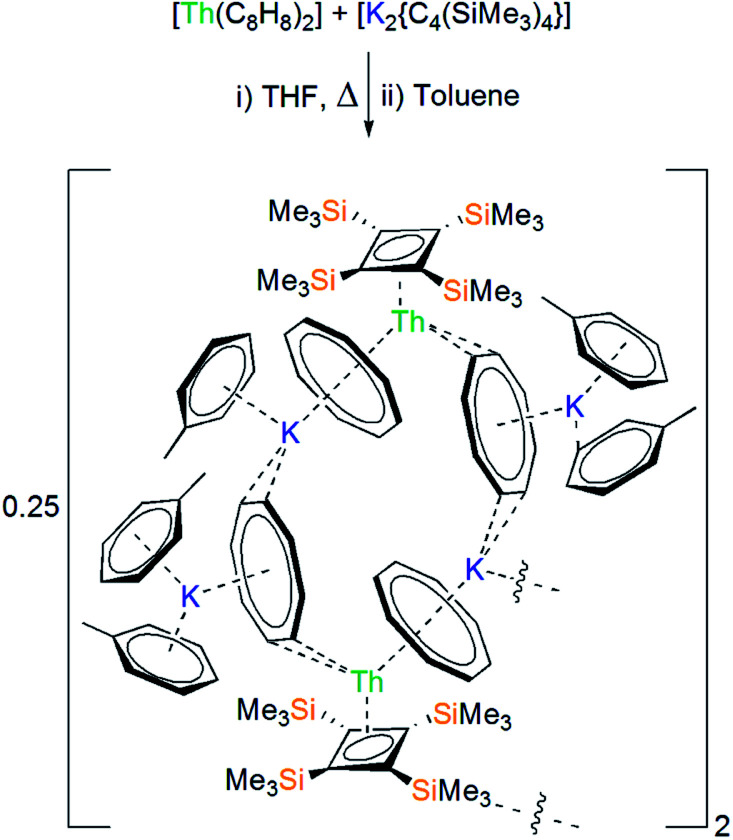
Synthesis of **1** from [Th(η^8^-C_8_H_8_)_2_] and [K_2_{C_4_(SiMe_3_)_4_}].

Condensation of THF onto a cold (−196 °C) solid mixture of [Th(η^8^-C_8_H_8_)_2_] and [K_2_{C_4_(SiMe_3_)_4_}], followed by thawing and heating for two hours, led to the formation of a bright orange solution. After work-up, recrystallisation from toluene afforded bright orange crystals, the colour of which assures the presence of thorium(iv), formulated as the co-complex [{Th(η^4^-C_4_[SiMe_3_]_4_)(μ-η^8^-C_8_H_8_)(μ-η^2^-C_8_H_8_)(K[C_6_H_5_Me]_2_)}_2_{K(C_6_H_5_Me)}{K}] (**1**) in 78% isolated yield (Scheme 1).^17^ Interestingly, **1** does not appear to react further if an additional equivalent of [K_2_{C_4_(SiMe_3_)_4_}] is added. The coordinated toluene molecules in **1** do not de-coordinate when **1** is placed under dynamic vacuum, but they do exchange with benzene when **1** is dissolved in this solvent.

### Crystallographic characterisation

**Fig. 1:**
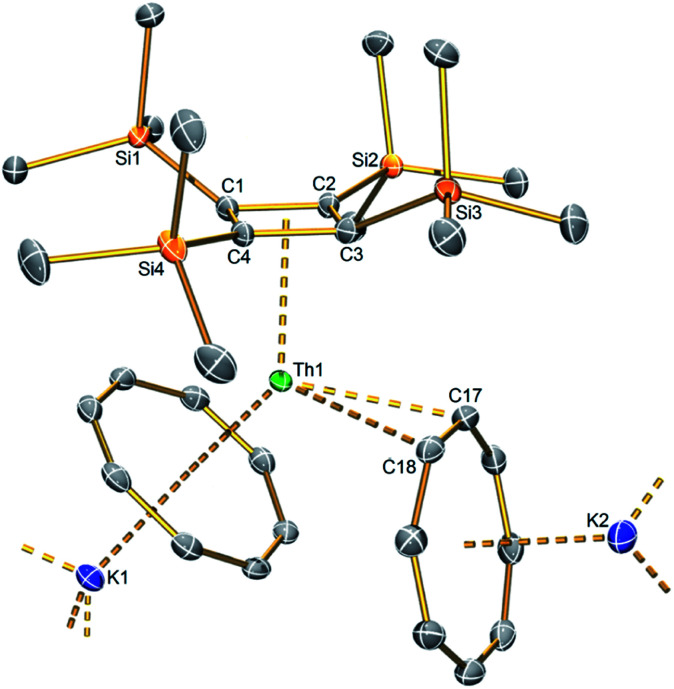
Solid state structure of the heteroleptic thorocene unit in **1** at 100 K with displacement ellipsoids set to 40%. Hydrogen atoms, the five coordinated toluene, and lattice toluene molecules omitted for clarity. Only one of the two [Th{η^4^-C_4_(SiMe_3_)_4_}(μ-η^8^-C_8_H_8_)(μ-η^2^-C_8_H_8_)(K)_2_] fragments from the asymmetric unit is shown; they are both very similar to each other, differing principally only in the varied multi-hapto coordination of toluene solvent and cyclooctatetraene and agostic-type trimethylsilyl interactions.

The solid-state structure of **1** was determined by X-ray diffraction (Fig. 1). Complex **1** crystallises with two [Th{η^4^-C_4_(SiMe_3_)_4_}(μ-η^8^-C_8_H_8_)(μ-η^2^-C_8_H_8_)(K)_2_] units in the crystallographic asymmetric unit, rendered inequivalent to each other by the nature of K-coordination environments: K4-(η^6^-C_6_H_5_Me)(η^8^-C_8_H_8_)(η^2^-C_8_H_8_); K3-(η^6^-C_6_H_5_Me)(η^1^-C_6_H_5_Me)(η^8^-C_8_H_8_); K2-(η^6^-C_6_H_5_Me)(η^6^-C_6_H_5_Me)(η^8^-C_8_H_8_); K1-(η^8^-C_8_H_8_)(η^2^-C_8_H_8_)(κ^1^-MeSiMe_2_). The latter interactions double-up the asymmetric unit resulting in a tetrathorium aggregate overall.^17^ In more detail, the salient structural features of each thorium-containing unit are η^4^-coordination of the {C_4_(SiMe_3_)_4_}^2−^ dianion and two {C_8_H_8_}^2−^ ligands which are η^8^-and η^2^-coordinated, the latter of which is highly unusual for a {C_8_H_8_}^2−^ ligand. Thus, the coordination geometry at each thorium ion resembles that of a bent C_4_/C_8_-metallocene with a coordinated alkene, the latter being a rare interaction for lanthanides^19^ and unknown in actinide chemistry, with the closest example for actinides being the reduced 1,2-ethanediide [{(η^8^-C_8_H_4_[SiPr^i^_3_]_2_)(η^5^-C_5_Me_5_)U}_2_(μ-η^2^-η^2^-C_2_H_4_)].^20^

The average Th–C(η^4^-C_4_(SiMe_3_)_4_) distances are 2.651(9) and 2.649(9) Å for Th1 and Th2, respectively; ∼0.14 Å greater than analogous value found for **A** (2.513(17) Å), though when considering the 3σ-criterion this difference falls to 0.06 Å.^8^ The latter is close to the 0.05 Å difference in the ionic six-coordinate radii of thorium(iv) (0.94 Å) and uranium(iv) (0.89 Å).^21^ Interestingly, the Th–C(η^4^-C_4_(SiMe_3_)_4_) distances span quite different ranges at Th1 and Th2, being 2.627(3)–2.672(3) and 2.557(3)–2.739(3) Å, respectively, which overall is a larger range of variance than **A** (2.477(11)–2.549(12) Å).^8^ These structural differences may reflect more polar Th–C bonding compared the U–C bonding in **A**, but may also reflect that the Th1 thorocene unit is more restricted within the tetrathorium motif than the Th2 thorocene unit and the coordination environments of **A** and **1** are rather different. However, for a firmer comparison, the Th–C(η^5^-C_5_Me_5_) distances in the bent thorium–pentamethylcyclopentadienyl–cyclooctatetraenyl metallocene [{(η^8^-C_8_H_8_)(η^5^-C_5_Me_5_)Th}_2_(μ-η^3^-η^3^-C_8_H_8_)] (**B**) range from 2.813(6)–2.864(6) Å, and average 2.843(8) Å.^22^ This is evidently greater than the average Th–C(η^4^-C_4_(SiMe_3_)_4_) distance in **1**, reflecting the formal dianionic charge on the cyclobutadienyl ligand in **1**, *versus* the mono anionic charge of the pentamethylcyclopentadienyl ligand in **B**. The average cyclobutadienyl C_4_-ring C–C distances in **1** are identical at 1.481(8) Å, and statistically indistinguishable from the analogous metric for **A** (1.488(17) Å).^8–10^

The displacement of the cyclobutadienyl silyl substituents in **1**, out of the plane of the C_4_-ring away from the respective thorium centres, is notable due to their asymmetry. Opposing silyl groups are displaced by similar distances to each other, but each pair is quite different to the other. At Th1, Si1/Si3 and Si2/Si4 pairs are displaced by 0.839(9)/0.830(12) and 0.233(9)/0.224(8) Å, respectively. At Th2, a larger range is found, with Si5/Si7 and Si6/Si8 displaced by 0.097(9)/0.297(8) and 0.678(6)/1.097(10) Å, respectively. In **A**, silyl displacements are also observed, but over a narrower range (0.452(3)–0.566(3) Å).^8^ This most likely can be attributed to the presence of three symmetrically coordinated κ^3^-borohydrides at uranium in **A** whereas in **1** the (η^8^-C_8_H_8_)(η^2^-C_8_H_8_) arrangement is asymmetric.

The average Th–C(η^8^-C_8_H_8_) distances are typical of such interactions, at 2.787(12) and 2.765(17) Å for Th1 and Th2, respectively,^1,4*e*,22^ and are unsurprisingly longer than the average Th–C(η^4^-C_4_(SiMe_3_)_4_) distances within **1**. For Th1 and Th2, the average Th–C(η^2^-C_8_H_8_) distances are 2.741(10) and 2.788(9) Å, respectively. Interestingly, the η^2^-C_8_H_8_ interaction appears to induce a lengthening of the η^2^-C–C distances (1.441(4) and 1.427(4) Å) compared to the rest of the C_8_-rings (1.397(4)–1.418(4) and 1.398(4)–1.414(4) Å, respectively); whilst there is some overlap in these distances by the 3σ-criterion it is certainly the case that the range for the η^2^-C–C distances sits at the top end of these C–C bond distances, hinting at depletion of C–C bond electron density.

There are few examples of structurally characterised metal-η^2^–cyclooctatetraenyl interactions,^22,23^ but they usually involve small, very polarising metals such as lithium and magnesium. The normal trend for cyclooctatetraenyl bonding is that the hapticity increases with increasing metal size,^24^ but when lower η values are observed they are overwhelmingly η^4^ butadienyl-like or η^3^ allyl-like. However, **1** exhibits thorium, one of the larger metals in the periodic table with the η^2^ alkene-like coordination mode. The C–C distances of the C_8_H_8_ unit are not obviously perturbed by η^2^-coordination to lanthanum in the complex [(μ-η^8^:η^2^-C_8_H_8_)La{η^5^-C_5_H_3_(SiMe_3_)_2_}(μ-η^8^:η^8^-C_8_H_8_)La{η^5^-C_5_H_3_(SiMe_3_)_2_}_2_][K(18-crown-6)],^25^ but would appear to be in the case of **1**, when η^2^-coordinated to thorium.

The Cnt(η^8^-C_8_H_8_)-Th-Cnt(η^4^-C_4_(SiMe_3_)_4_) (Cnt = centroid of the C_*n*_-ring) angles within **1** deviate significantly from linearity, at 139.3° for Th1 and 136.9° for Th2. These values are similar to the Cnt-Th-Cnt angle of 144.2° for the bent thorocene complex [Th(η^8^-C_8_H_8_)_2_(Me_4_phen)] (Me_4_phen = 3,4,7,8-tetramethyl-1,10-phenanthroline) and **B** (132.11°).^4*e*,22^ The C_8_⋯K distances are unexceptional.^26^

### Spectroscopic analyses

The ^1^H NMR spectrum of **1** in C_6_D_6_ exhibits a sharp resonance (full width half maximum = 1.98 Hz) at 0.74 ppm, corresponding to the trimethylsilyl substituents of the cyclobutadienyl ligands. Additionally, two equally broad resonances from the cyclooctatetraenyl hydrogen atoms are exhibited at 5.71 and 6.47 ppm, with full width half maxima values of 26.5 and 28.7 Hz, respectively. This indicates that two distinct cyclooctatetraenyl environments are present in solution, a situation which is also observed for the crude post-reaction product dissolved in THF (Fig. S8†). Unfortunately, variable-temperature and DOSY experiments (arenes or THF) were not possible due to **1** precipitating on standing or cooling or showing signs of decomposing upon heating for prolonged periods. It seems likely that the more downfield resonance corresponds to the {η^8^-C_8_H_8_}^2−^ dianion,^18,22^ since this is close to the value of 6.50 ppm for thorocene. The upfield resonance is more in the region associated with neutral tetraolefinic C_8_H_8_, and if the η^2^-coordination of the [C_8_H_8_]^2−^ dianion to thorium is maintained in solution, this would be expected to result in C–C π-bond relocalisation to some extent,^27^ as suggested by the structural data for **1**. We note that the upfield resonance is shifted slightly from [K_2_(C_8_H_8_)] (5.76 ppm),^28^ which is not unexpected given **1** is a co-complex.

The optical spectrum of **1** in toluene is dominated by a broad charge transfer band, which spans from ∼18 000 cm^−1^ into the UV (Fig. S11†). This is consistent with the intense colour of **1** in solution and solid phases, and is modelled well by TD-DFT calculations (*vide infra*).

### Computational analyses

In order to further understand the nature of the bent, heteroleptic thorocene unit in **1** we undertook DFT calculations.^17^ With the whole tetrathorium assembly of **1** being unrealistic to compute, we focussed on a discrete [Th{η^4^-C_4_(SiMe_3_)_4_}(η^8^-C_8_H_8_)] unit. However, geometry optimisation converges to a less bent structure in the gas phase, with a computed Cnt(η^8^-C_8_H_8_)-Th-Cnt(η^4^-C_4_(SiMe_3_)_4_) angle of 160.5°. A single point energy calculation on the crystallographic coordinates returns a total bond energy that is ∼332 kcal mol^−1^ (3.6%) destabilised compared to the geometry optimised total bond energy. This clearly represents the upper bound at most, since the single point energy coordinates have not been allowed to relax in any way in this scenario, but is an instructive parameter since it suggests that the Th-η^2^-C_8_H_8_ interaction brings significant stabilisation to **1** since its inclusion clearly compensates for the energetic penalty of bending the [Th{η^4^-C_4_(SiMe_3_)_4_}(η^8^-C_8_H_8_)] unit to accommodate it.

**Fig. 2:**
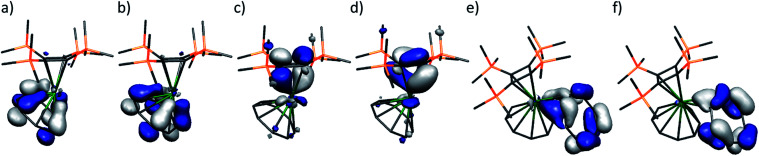
Frontier molecular orbitals of computational models **1′** and **1′′**. (a) HOMO−3 δ-bond (164, −5.093 eV) of **1′**, (b) HOMO−2 δ-bond (165, −5.036 eV) of **1′**, (c) HOMO−1 π-bond (166, −4.218 eV) of **1′**, (d) HOMO π-bond (167, −4.119 eV) of **1′**, (e) HOMO−1 π-bond (195, 3.018 eV) of **1′′**, (f) HOMO σ-bond (196, 3.145 eV) of **1′′**. The positive energies of the HOMO and HOMO−1 of **1′′** reflect the formal 2− charge applied to the system, but the electrons are clearly not detached. Hydrogen atoms are omitted for clarity.

To provide a benchmark, we first describe pertinent aspects of the electronic structure of the [Th{η^4^-C_4_(SiMe_3_)_4_}(η^8^-C_8_H_8_)] (**1′**) unit from the single point energy calculation on the crystallographic coordinates derived from **1** (Fig. 2a–d). The HOMO and HOMO−1 of **1′** are two quasi-degenerate (−4.119 and −4.218 eV, respectively) thorium–cyclobutadienyl π-bonds each composed of 22 : 78% Th : C character, deriving from the *ψ*_2_ and *ψ*_3_ π-symmetry molecular orbitals of cyclobutadienyl and thorium (6d : 5f – 69 : 31%). The HOMO−2 and HOMO−3 are two quasi-degenerate (−5.036 and −5.093 eV) thorium–cyclooctatetraenyl δ-bonds each composed of 19 : 81% Th : C character, deriving from the *ψ*_4_ and *ψ*_5_ δ-symmetry molecular orbitals of cyclooctatetraenyl and thorium orbitals (6d : 5f – 92 : 8%). Whilst the thorium percentage character contributions to the molecular orbitals of **1′** are quite similar for both ring types, and thorium utilises mainly 6d-orbital character in its bonding generally, substantially more 5f character is involved with the smaller cyclobutadienyl ring compared to the more expansive cyclooctatetraenyl ring; this may reflect the more angular character of 5f-compared to 6d-orbitals.^2*b*,6^ When compared to **A**,^8^ the thorium contributions to the thorium–cyclobutadienyl π-bonds in **1′** are ∼8% lower than for uranium (U : C 30 : 70%) but, whilst the thorium 5f contribution is *ca.* half that found in **A** (6d : 5f – 33 : 67%) it is surprisingly high for thorium.

The Th–C Mayer bond orders for **1′** average 0.50 and 0.36 for the Th–C(η^4^-C_4_(SiMe_3_)_4_) and Th–C(η^8^-C_8_H_8_) interactions, respectively, and sum to 2.0 and 2.84, respectively. The C–C bond orders are 1.0 and 1.2 for the cyclobutadienyl and cyclooctatetraenyl rings, and the computed charges of +1.9 (Th), −1.7 (C_4_), and −2.3 (C_8_) perhaps suggest a greater engagement of the cyclobutadienyl ligand to thorium than the cyclooctatetraenyl ring, in-line with the Mayer bond orders.

Since DFT calculations produce an orbital-based bonding picture, we examined the bonding topology in **1′** with the QTAIM method. This reveals computed average *ρ*/∇^2^*ρ*/*H* (energy)/*ε* (ellipticity) 3,−1 bond critical point values for the Th–C(η^4^-C_4_(SiMe_3_)_4_) and Th–C(η^8^-C_8_H_8_) interactions in **1′** of 0.05/0.08/−0.02/0.13 and 0.03/0.08/−0.02/0.49; these data in gross terms compare well to computed data for [U(η^5^-C_5_H_5_)_4_],^29^ though for the cyclobutadienyl component they indicate slightly weaker Th–C interactions than in **A**.^8^

With the bonding within **1'** benchmarked, we now consider the bonding of [Th{η^4^-C_4_(SiMe_3_)_4_}(η^8^-C_8_H_8_)(η^2^-C_8_H_8_)]^2−^ (**1′′**). To enable meaningful comparisons to be made between **1′** and **1′′** this analysis is on a single point energy calculation on crystallographic coordinates, though the analysis is anyway qualitative due to the necessity to introduce the formal 2− charge for **1′′** since inclusion of charge-balancing potassium ions and toluene solvent in a realistic model was impracticable given the pseudo-periodic nature of **1**. The most obvious effect of binding of the η^2^-C_8_H_8_ ligand to thorium is that the Th–C(η^4^-C_4_(SiMe_3_)_4_) and Th–C(η^8^-C_8_H_8_) Mayer bond orders decrease to 0.33 and 0.29, respectively. In this context the Th–C(η^2^-C_8_H_8_) average Mayer bond order of 0.35 is significant. Inspection of the molecular orbital manifold reveals that in **1′′** the thorium–cyclobutadienyl and –cyclooctatetraenyl bonding interactions are far more mixed than in **1'**, across HOMOs −2 to −5. The HOMO and HOMO−1 account for the Th-η^2^-C_8_H_8_ interaction (Fig. 2d and e). With respect to the coordinated C=C unit 2p π-symmetry orbitals, the HOMO is an in-phase in-plane σ-donation, whereas HOMO−1 is an out-of-phase in-plane π-donation. These two orbitals correspond to combinations from the doubly occupied *ψ*_4_ and *ψ*_5_ δ-symmetry molecular orbitals of cyclooctatetraenyl into vacant thorium 6d orbitals.

QTAIM analysis finds average *ρ*/∇^2^*ρ*/*H* (energy)/*ε* (ellipticity) 3,−1 bond critical point values for the two Th–C(η^2^-C_8_H_8_) interactions of 0.04/0.08/−0.02/0.31; these are in between the Th–C(η^4^-C_4_(SiMe_3_)_4_) and Th–C(η^8^-C_8_H_8_) values for **1′**. When taking all these data together, it is clear that the Th–C(η^2^-C_8_H_8_) interaction is significant.

The calculations on **1′′** provide a basis to probe the experimental spectroscopic data of **1**. The principal absorbances in the 18 000–24 000 cm^−1^ region from a TD-DFT calculation^17^ on **1′′** correspond to transitions into the LUMO, which is the *ψ*_4_ δ-symmetry molecular orbital of cyclobutadienyl, from the thorium–cyclobutadienyl and –cyclooctatetraenyl bonding molecular orbitals. The transition that gives **1** its striking orange colour is LMCT at 24 270 cm^−1^ from the η^2^-coordinated cyclooctatetraenyl ligand to vacant thorium 6d orbitals. As expected, TD-DFT calculations on [Th{η^4^-C_4_(SiMe_3_)_4_}(η^8^-C_8_H_8_)] (crystallographic or geometry optimised coordinates), where the additional η^2^-(C_8_H_8_)^2−^ dianion ligand is omitted, reveals transitions principally involving *ψ*_2/3_ transitions to *ψ*_4_ for the Th–C_4_ interactions in the 20 000–23 000 cm^−1^ region, then above 30 000 cm^−1^ transitions involve Th–C_8_ (*δ*) to Th–C_4_ (*δ*) transitions, but the 23 000–30 000 cm^−1^ region is essentially void of transitions, with any oscillator strengths being 2–4 orders of magnitude lower in intensity.

## Summary and conclusions

To conclude, although the {C_4_(SiMe_3_)_4_}^2−^ dianion is too reducing for polar thorium tetrachloride, reacting to produce intractable precipitates and the neutral cyclobutadiene {C_4_(SiMe_3_)_4_}, we find that less polar thorocene is an effective starting material from which to prepare a thorium–cyclobutadienyl complex, which is notable since actinocenes are usually regarded as thermodynamic sinks. Within this new type of thorocene co-complex is the unprecedented heteroleptic thorocene dianion unit [Th{η^4^-C_4_(SiMe_3_)_4_}(η^8^-C_8_H_8_)(η^2^-C_8_H_8_)]^2−^, which exhibits an exceedingly unusual thorium–η^2^-C_8_H_8_ alkene-type interaction that is composed of σ- and π-symmetry alkene-like donation from in-plane in- and out-of-phase C=C p-orbital combinations to formally vacant thorium 6d orbitals. Complementary spectroscopic and computational characterisation data provide evidence which suggests that LMCT from the (η^2^-C_8_H_8_)^2−^ ligand to vacant thorium 6d orbitals accounts for the vivid orange colour of **1**. The combination of cyclobutadienyl and cyclooctatetraenyl ligands bound to one thorium showcases the flexible nature of thorium–ligand bonding, not only in terms of π *vs.* δ bonding to carbocyclic rings of different sizes, but also the surprising use of 5f-as well as 6d-orbital character to support the bonding to small aromatic rings. Whilst the general picture of thorium–ligand bonding being more polar than uranium is reflected in our calculations, they also reveal surprisingly high levels of thorium 5f orbital character in the bonding, which is not entirely in-line with the traditional of thorium and uranium displaying 6d *vs.* 5f orbital character, respectively.

## Conflicts of interest

There are no conflicts to declare.

## Supplementary Material

SC-011-D0SC02479A-s001

SC-011-D0SC02479A-s002
