# Supplementary material for: Heteroleptic actinocenes: a thorium(iv)–cyclobutadienyl–cyclooctatetraenyl–di-potassium-cyclooctatetraenyl complex
Source: Chem Sci. 2020 Jun 10;11(26):6789–94. doi: 10.1039/d0sc02479a (PMC8159314; doi:10.1039/d0sc02479a)
Supplement: SC-011-D0SC02479A-s001 [file SC-011-D0SC02479A-s001.pdf]

Electronic Supplementary Material (ESI) for Chemical Science.
This journal is © The Royal Society of Chemistry 2020

- Electronic Supplementary Information -

Heteroleptic Actinocenes: A Thorium(IV)-Cyclobutadienyl-Cyclooctatetraenyl-Di-Potassium-Cyclooctatetraenyl Complex

Josef T. Boronski, Ashley J. Wooles, and Stephen T. Liddle*

Department of Chemistry, The University of Manchester, Oxford Road, Manchester, M13 9PL, UK

E-mail: steve.liddle@manchester.ac.uk

Experimental Details

General

All manipulations were carried out using Schlenk techniques, or an MBraun UniLab glovebox, under an atmosphere of dry nitrogen. Solvents were dried by passage through activated alumina towers, dried with NaK_2 and degassed before use. All solvents were stored over NaK_2. Deuterated solvent was distilled from NaK_2, degassed by three freeze-pump-thaw cycles and stored under nitrogen. ^{1}H, ^{13}C and ^{29}Si NMR spectra were recorded on a Bruker 400 spectrometer operating at 400.2, 100.62 and 79.49 MHz respectively; chemical shifts are quoted in ppm and are relative to $SiMe_4$. FTIR spectra were recorded on a Bruker Alpha spectrometer with Platinum-ATR module. UV/Vis/NIR spectra were recorded on a Perkin Elmer Lambda 750 spectrometer using a 1 mm path length cuvette loaded in an MBraun UniLab glovebox and run versus toluene reference solvent. Elemental microanalyses were carried out by Mr Martin Jennings at the Micro Analytical Laboratory, School of Chemistry, The University of Manchester. The compounds $[K_2\{C_4(SiMe_3)_4\}]$, $[Th(C_8H_8)_2]$, and $[ThCl_4(THF)_{3.5}]$ were prepared as described previously.[1-3]

Reaction of [ThCl$_4$(THF)$_{3.5}$] with [K$_2${C$_4$(SiMe$_3$)$_4$}]

C_6D_6 (0.5 ml) was added to a solid mixture of [ThCl$_4$(THF)$_{3.5}$] (15 mg, 0.024 mmol) and [K$_2${C$_4$(SiMe$_3$)$_4$}] (20 mg, 0.048 mmol) at room temperature. The red solution turned paler in colour, and deposition of a dark grey precipitate was observed over a period of 24 hours. The ^{1}H and ^{29}Si NMR spectra recorded for this solution revealed formation of {C$_4$(SiMe$_3$)$_4$}, in addition to unreacted [K$_2${C$_4$(SiMe$_3$)$_4$}] and other unidentified species.[1,4] The dark grey precipitate was found to be insoluble, even in boiling THF.

Synthesis of [{Th(η^4-C$_4$[SiMe$_3$]$_4$)(μ-η^8-C$_8$H$_8$)(μ-η^2-C$_8$H$_8$)(K[C$_6$H$_5$Me]$_2$)}$_2${K(C$_6$H$_5$Me)}{K}] (1)

To a Young's ampoule equipped with a glass stirrer bar was added a solid mixture of [Th(C$_8$H$_8$)$_2$] (50 mg, 0.14 mmol) and [K$_2${C$_4$(SiMe$_3$)$_4$}] (48 mg, 0.17 mmol). THF (5 ml) was condensed into the vessel *in vacuo* at −196 °C. The red mixture was allowed to warm to room temperature, then heated at 70 °C for 2 hours with stirring, turning bright orange in colour. Subsequently, the volatiles were removed *in vacuo* and the oily orange solid was washed with pentane (3 x 5 ml). Residues were extracted into toluene (5 ml) and filtered. Next, the solution was concentrated to approximately 3 ml and transferred to a vial inside a glovebox. Slow evaporation of the solvent over 48 hours at room temperature led to the formation of a large crop of bright orange crystals of **1**. Yield: 97 mg, 78%. Single crystals of **1** suitable for X-ray diffraction experiments were obtained by slow evaporation of a concentrated toluene solution in a glovebox. Anal. Calcd for $C_{99}H_{144}K_4Si_8Th_2$: C, 54.56; H, 6.66. Found: C, 54.39; H, 6.46. ^{1}H NMR (C_6D_6, 298 K): δ 7.14 (m, C$_6$*H*$_5$Me), 7.03 (m, C$_6$*H*$_5$Me), 6.47 (br s, 16H, C$_8$*H*$_8$), 5.71 (br s, 16H, C$_8$*H*$_8$), 2.11 (s, C$_6$H$_5$(C*H*$_3$)), 0.74 (s, 72H, -Si(C*H*$_3$)$_3$). ^{13}C{^{1}H} NMR (C_6D_6, 298 K): δ 141.03 (*C*$_4$(SiMe$_3$)$_4$), 137.90 (*C*$_6$H$_5$Me), 129.34 (*C*$_6$H$_5$Me), 128.57 (*C*$_6$H$_5$Me), 125.70 (*C*$_6$H$_5$Me), 97.7 (br, *C*$_8$H$_8$), 21.43 (s, C$_6$H$_5$(*C*H$_3$)), 4.47 (-Si(*C*H$_3$)$_3$). The

second C_8H_8 resonance could not be observed, which is attributed to a combination of **1** not being soluble enough, and the resonance being too broad to observe at the concentration level that was achievable, or the resonance could be obscured by the benzene resonance. Variable temperature experiments were precluded due to **1** precipitating on cooling or signs of decomposition at elevated temperatures over prolonged periods of time (benzene, toluene, THF). $^{29}Si\{^1H\}$ NMR (C_6D_6, 298 K): δ −22.31. ATR-IR ν/cm^{-1}: 2948 (w), 2363 (w), 1304 (m), 1229 (s), 1181 (s), 1121 (s), 980 (s), 892 (w), 829 (m), 744 (m), 719 (m), 680 (m), 524 (m), 459 (m), 428 (w). UV-vis (toluene): λ_{max} nm (ε/mol^{-1}cm^{-1}): 458 (220), 859 (12), 1135 (9), 1669 (30), 2135 (57), 2339 (32), 2447 (107), 2680 (16), 2723 (22). Post-reaction crude product ^1H NMR (d_8-THF, 298 K): δ 6.05 (br s, 8H, C_8H_8), 5.67 (br s, 8H, C_8H_8), 0.27 (s, 36H, -Si(CH_3)$_3$). $^{13}C\{^1H\}$ NMR (d_8-THF, 298 K): δ 136.87 (C_4(SiMe$_3$)$_4$), 98.89 (C_8H_8), 98.56 (C_8H_8), 5.89 (-Si(CH_3)$_3$).

Solid State Structure

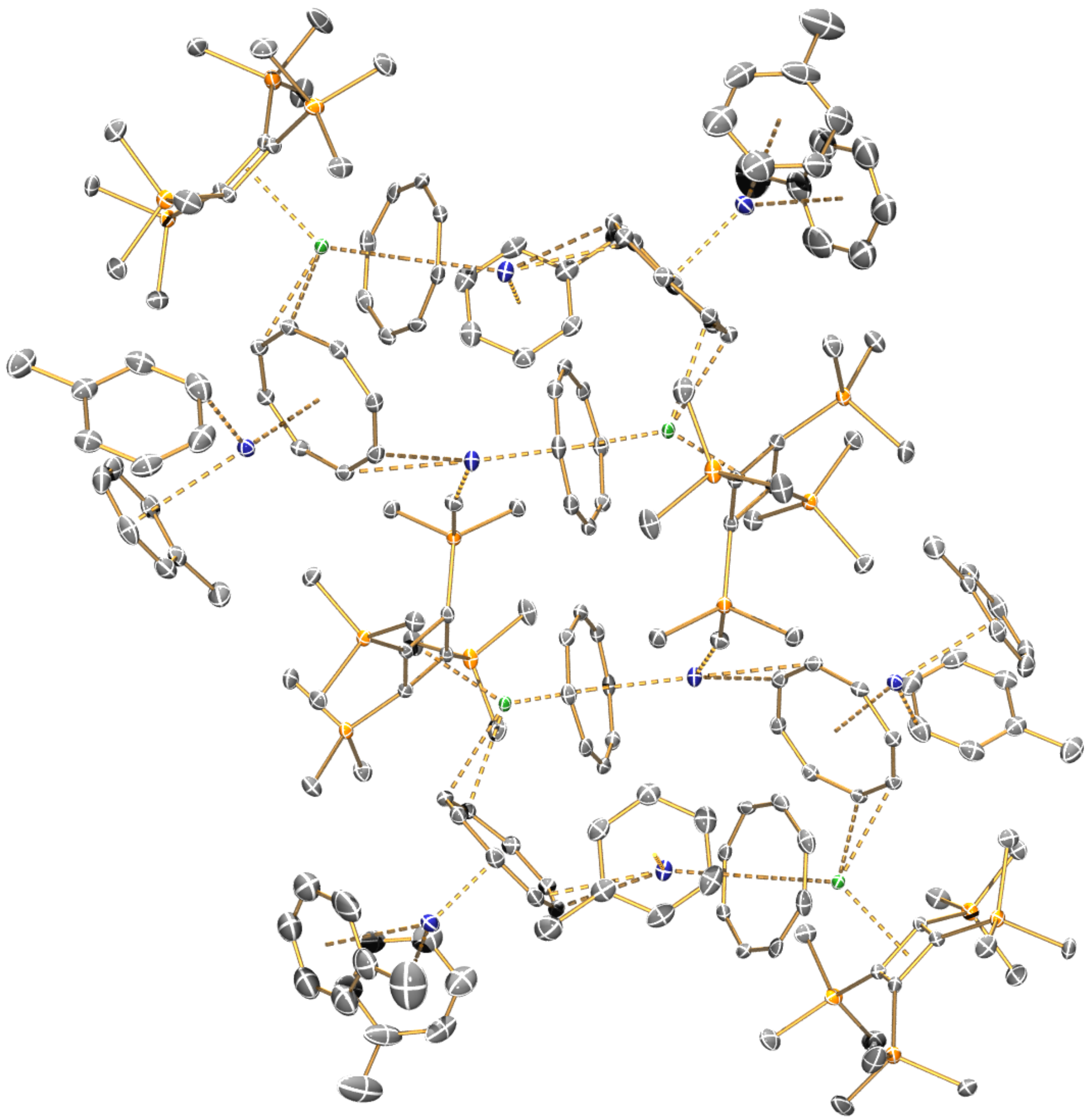

Figure S1. ***Molecular structure of the whole tetrathorium aggregate of 1 at 100 K. Displacement ellipsoids are set at 40% with hydrogen atoms and toluene molecules omitted for clarity. Atom key: thorium, green; potassium, navy blue; silicon, orange; carbon, gray.***

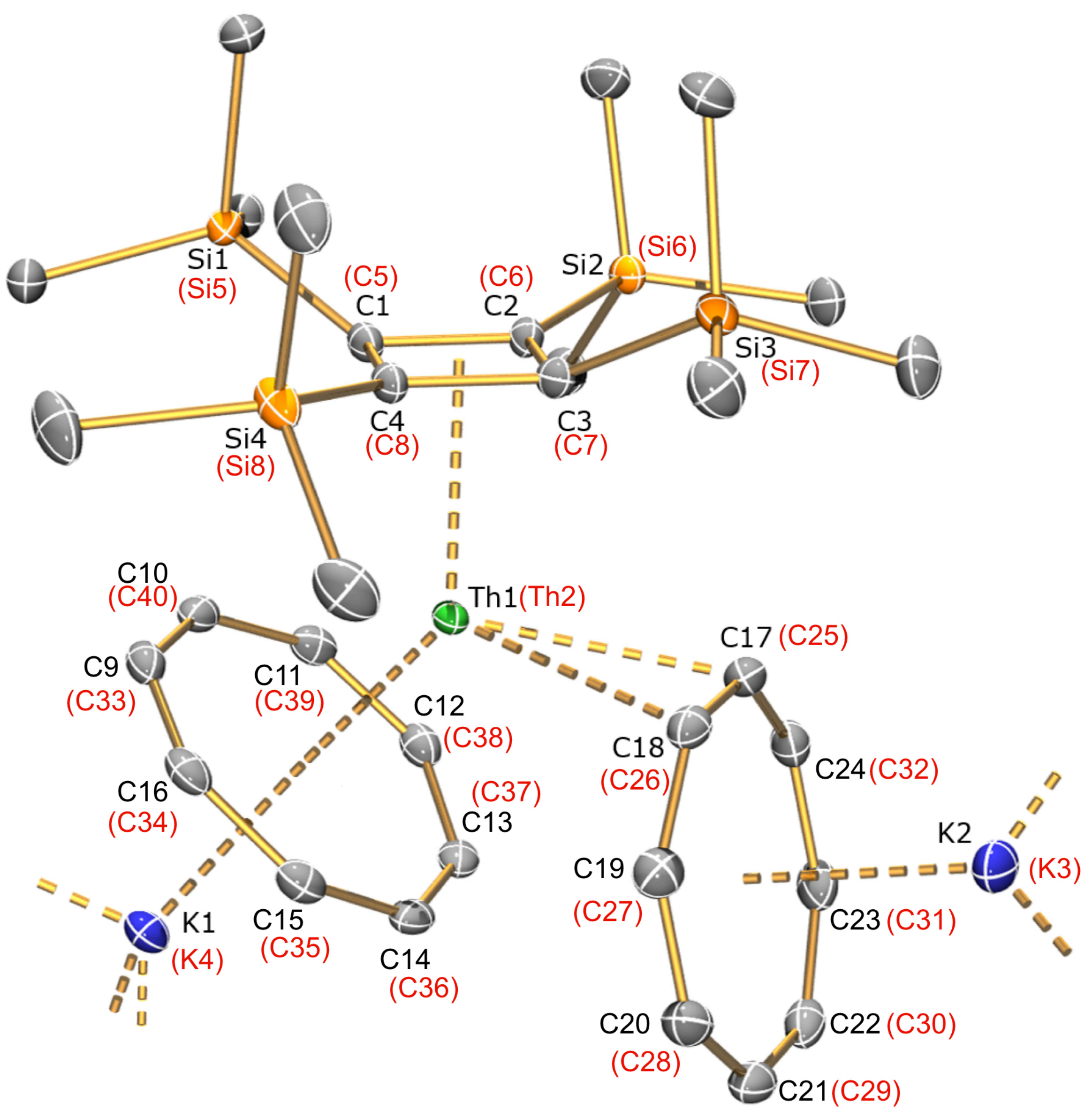

Figure S2. Molecular structure of one thorium-containing unit of 1 with atomic labels in black and red to differentiate the labelling of the two independent units in the crystallographic unit cell. Displacement ellipsoids are set at 40% with hydrogen atoms and toluene molecules omitted for clarity. Atom key: thorium, green; potassium, navy blue; silicon, orange; carbon, gray.

Table S1. Selected bond lengths (Å) and angles (º) within 1 (cnt = centroid, pln = plane).

Metric	Measurement	Metric	Measurement
**Th1-C(η^4-C_4R_4)**		**Th1-C(η^4-C_4R_4)**	
Th1-C1	2.627(3)	Th1-C5	2.650(3)
Th1-C2	2.658(3)	Th1-C6	2.739(3)
Th1-C3	2.672(3)	Th1-C7	2.650(3)
Th1-C4	2.647(3)	Th1-C8	2.557(3)
**Th1-C(η^2-C_8H_8)**		**Th2-C(η^2-C_8H_8)**	
Th1-C17	2.734(3)	Th2-C25	2.784(3)
Th1-C18	2.747(3)	Th2-C26	2.792(3)
**Th1-C(η^8-C_8H_8)**		**Th2-C(η^8-C_8H_8)**	
Th1-C9	2.861(3)	Th1-C33	2.827(9)
Th1-C10	2.840(3)	Th1-C34	2.795(8)
Th1-C11	2.824(3)	Th1-C35	2.767(9)
Th1-C12	2.798(3)	Th1-C36	2.791(11)
Th1-C13	2.811(3)	Th1-C37	2.799(10)
Th1-C14	2.802(3)	Th1-C38	2.770(9)
Th1-C15	2.786(3)	Th1-C39	2.801(10)
Th1-C16	2.792(3)	Th1-C40	2.870(10)
**C-C(η^8-C_8H_8)**		**C-C(η^2-C_8H_8)**	
C9-C10	1.414(4)	C17-C18	1.427(4)
C10-C11	1.413(4)	C18-C19	1.414(4)
C11-C12	1.414(4)	C19-C20	1.398(4)
C12-C13	1.406(4)	C20-C21	1.412(5)

C13-C14	1.405(4)	C21-C22	1.401(5)
C14-C15	1.420(4)	C22-C23	1.411(5)
C15-C16	1.414(4)	C23-C24	1.407(4)
C16-C9	1.409(4)	C24-C17	1.412(4)
C33-C34	1.402(6)	C25-C26	1.441(4)
C34-C35	1.405(6)	C26-C27	1.412(4)
C35-C36	1.409(6)	C27-C28	1.402(4)
C36-C37	1.409(6)	C28-C29	1.418(4)
C37-C38	1.408(6)	C29-C30	1.406(4)
C38-C39	1.408(6)	C30-C31	1.415(4)
C39-C40	1.411(5)	C31-C32	1.397(4)
C40-C33	1.408(6)	C32-C25	1.417(4)
**C-C(η^4-C_4R_4)**		**C-C(η^4-C_4R_4)**	
C1-C2	1.481(4)	C5-C6	1.458(4)
C2-C3	1.474(4)	C6-C7	1.467(4)
C3-C4	1.482(4)	C7-C8	1.503(4)
C4-C1	1.486(4)	C8-C5	1.496(4)
**C-Si**		**C-Si**	
C1-Si1	1.841(3)	C5-Si5	1.851(3)
C2-Si2	1.850(3)	C6-Si6	1.860(3)
C3-Si3	1.859(3)	C7-Si7	1.847(3)
C4-Si4	1.845(3)	C8-Si8	1.836(3)
**K1-C(η^8-C_8H_8)**		**K2-C(η^8-C_8H_8)**	
K1-C9	3.105(3)	K2-C17	2.953(3)

K1-C10	3.109(3)	K2-C18	2.955(3)
K1-C11	3.184(3)	K2-C19	3.050(3)
K1-C12	3.181(3)	K2-C20	3.050(3)
K1-C13	3.079(3)	K2-C21	2.993(3)
K1-C14	3.035(3)	K2-C22	2.986(3)
K1-C15	3.087(3)	K2-C23	3.042(3)
K1-C16	3.136(3)	K2-C24	3.055(3)
**K3-C(η^8-C_8H_8)**		**K4-C(η^8-C_8H_8)**	
K3-C25	2.936(3)	K4-C33	3.075(9)
K3-C26	2.913(3)	K4-C34	3.069(8)
K3-C27	3.002(3)	K4-C35	3.073(9)
K3-C28	3.008(3)	K4-C36	3.078(10)
K3-C29	2.964(3)	K4-C37	3.191(11)
K3-C30	2.965(3)	K4-C38	3.346(11)
K3-C31	2.993(3)	K4-C39	3.301(11)
K3-C32	2.973(3)	K4-C40	3.150(9)
**Cnt(η^8-C_8H_8)-Th1-Cnt(η^4-C_4R_4)**	139.31(12)	**Cnt(η^8-C_8H_8)-Th2-Cnt(η^4-C_4R_4)**	136.92(13)
**Si-pln<C1-C4>**		**Si-pln<C5-C8>**	
Si1-pln<C1-C4>	0.839(9)	Si5-pln<C5-C8>	0.097(9)
Si2-pln<C1-C4>	0.233(9)	Si6-pln<C5-C8>	0.678(6)
Si3-pln<C1-C4>	0.830(12)	Si7-pln<C5-C8>	0.297(8)
Si4-pln<C1-C4>	0.224(8)	Si8-pln<C5-C8>	1.097(10)

Spectroscopic Data

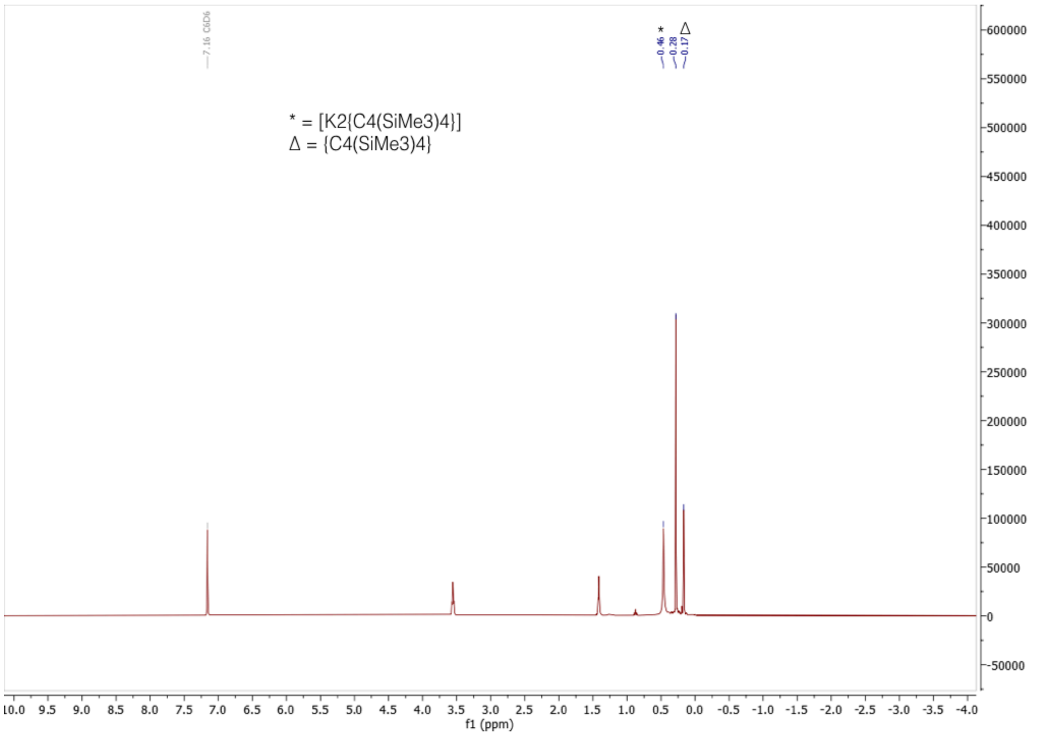

Figure S3. ***¹H NMR spectrum of the reaction of [ThCl₄(THF)₃.₅] with two equivalents of [K₂{C₄(SiMe₃)₄}] in C₆D₆.***

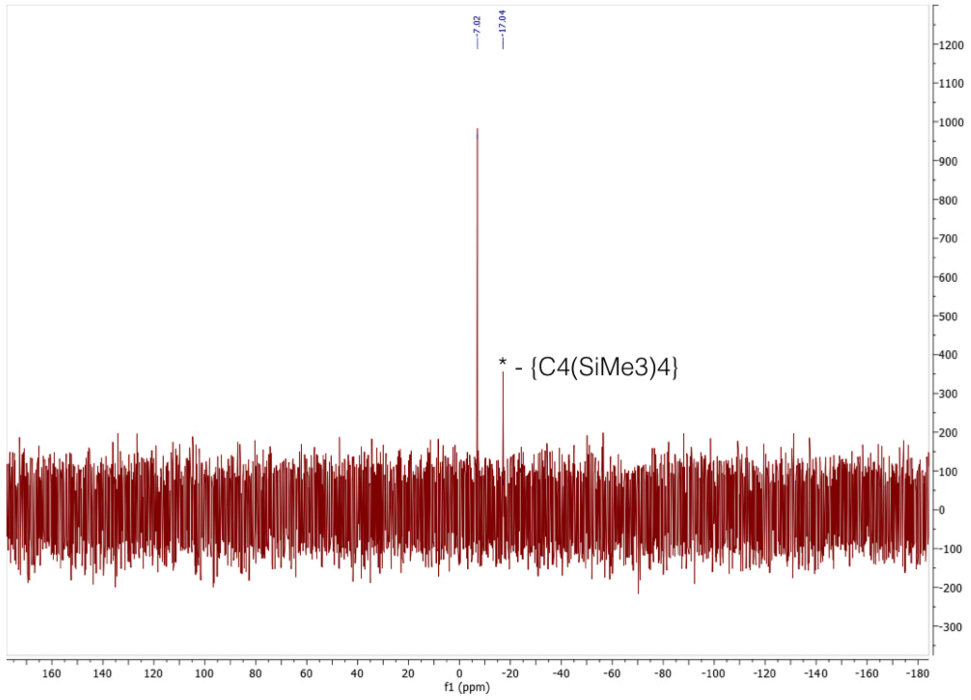

Figure S4. ***²⁹Si DEPT90 NMR spectrum of the reaction of [ThCl₄(THF)₃.₅] with two equivalents of [K₂{C₄(SiMe₃)₄}] in C₆D₆.***

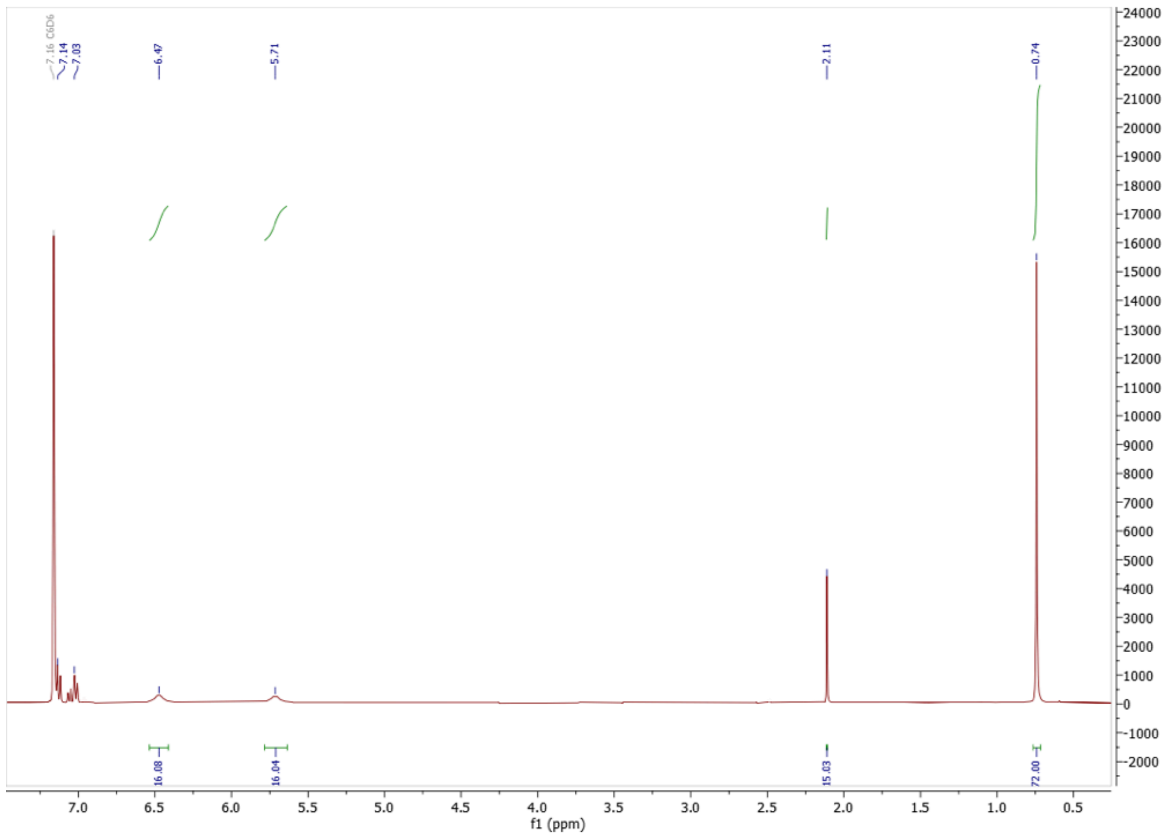

Figure S5. ***^{1}H NMR spectrum of 1 in C_6D_6.***

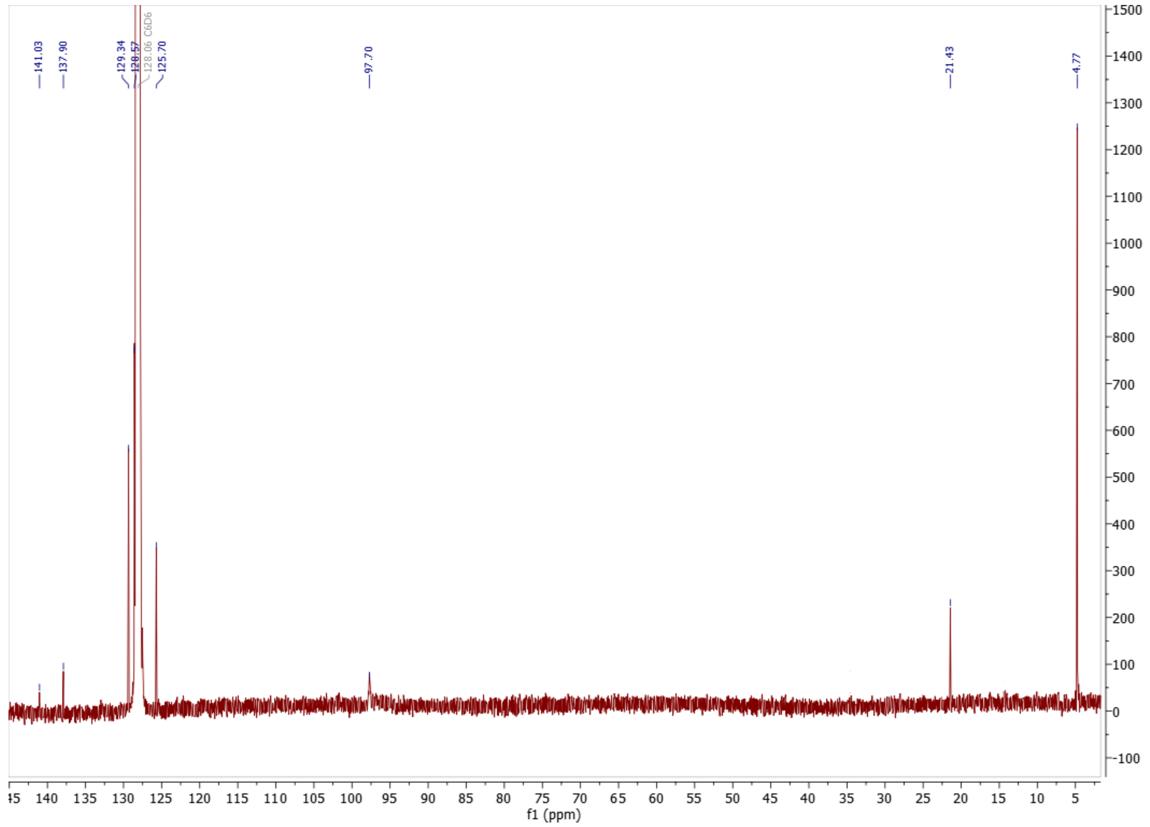

Figure S6. ***$^{13}C\{^{1}H\}$ NMR spectrum of 1 in C_6D_6.***

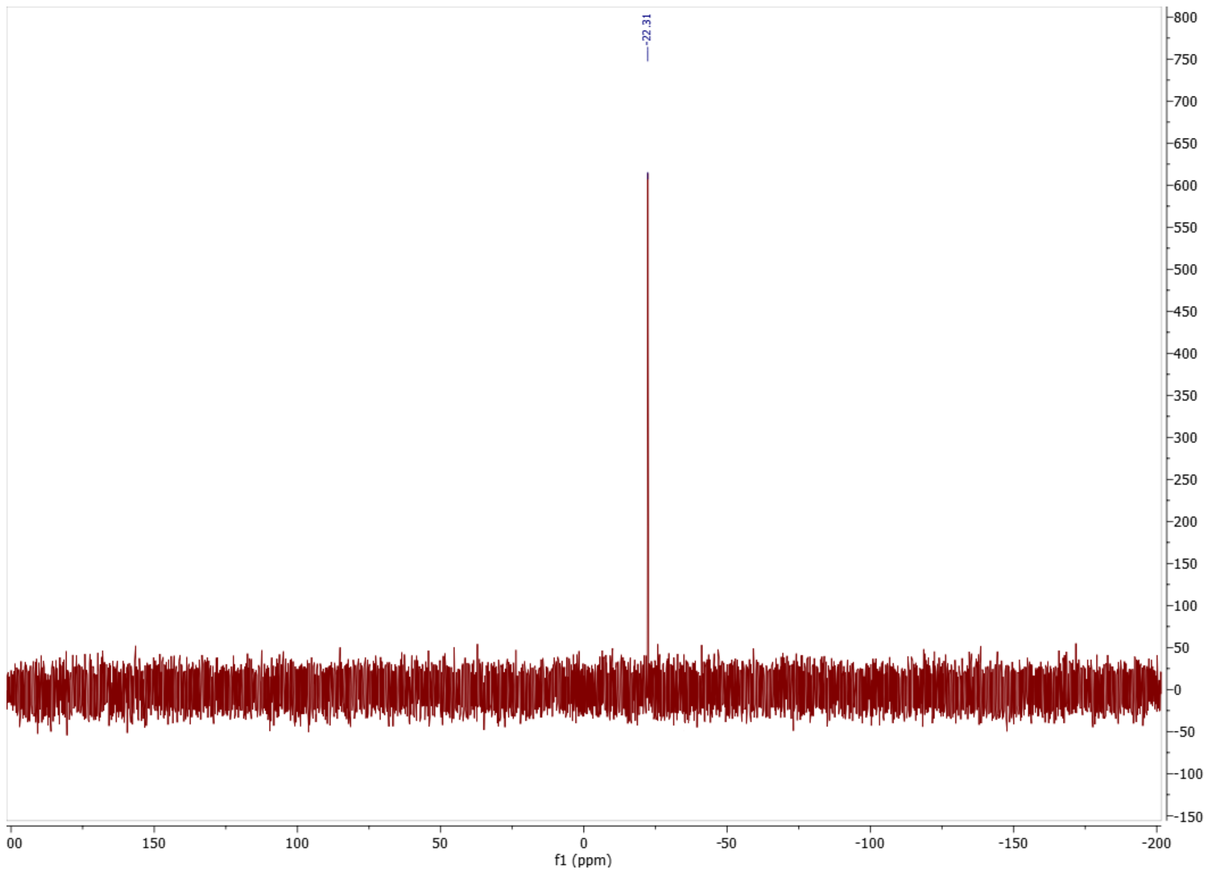

Figure S7. ***^{29}Si DEPT90 NMR spectrum of 1 in C_6D_6.***

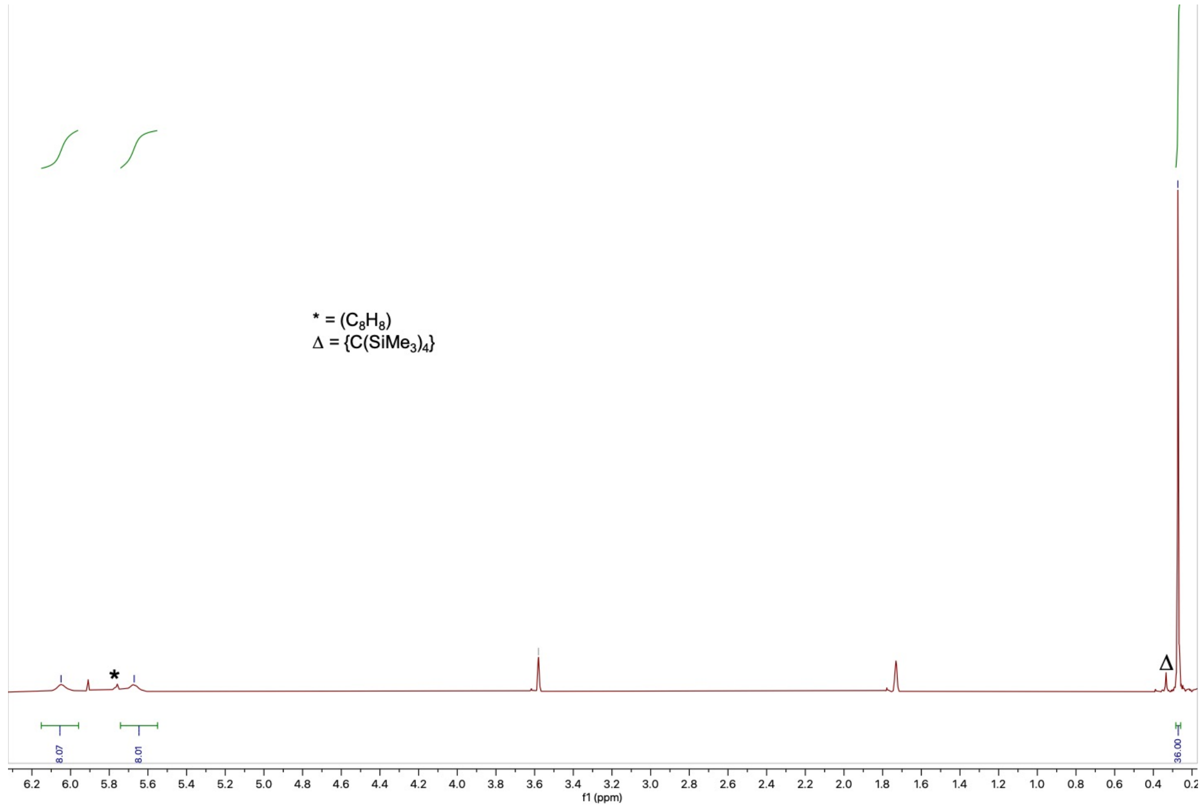

Figure S8 . ***^{1}H NMR spectrum of post-reaction crude product 1 in THF.***

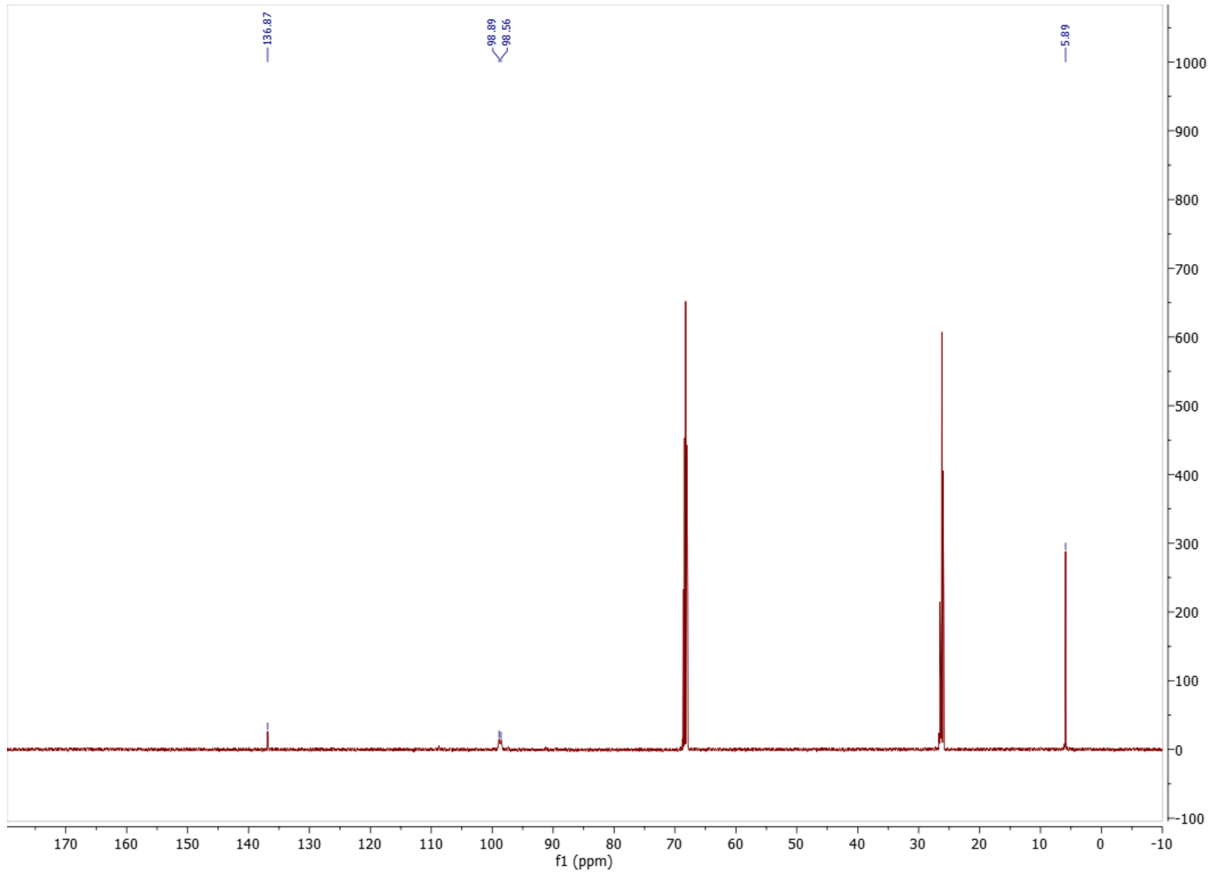

Figure S9. ***$^{13}C\{^{1}H\}$ NMR spectrum of post-reaction crude product 1 in THF.***

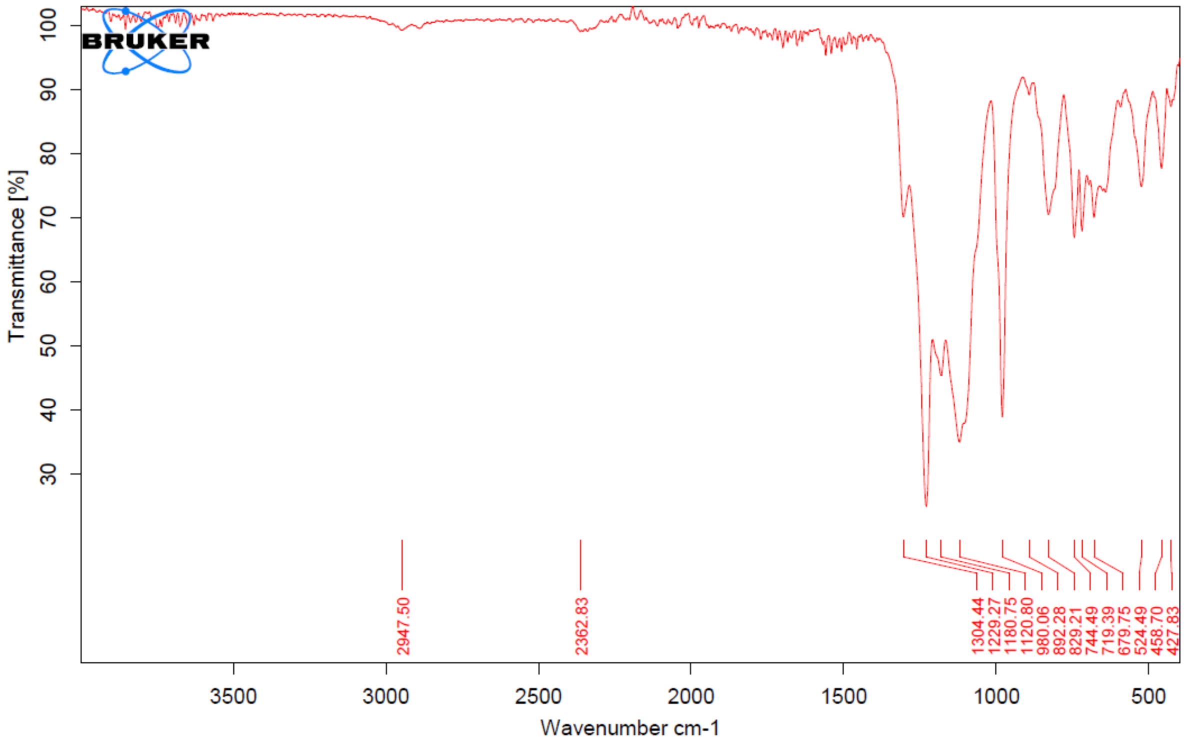

Figure S10. FTIR spectrum of 1.

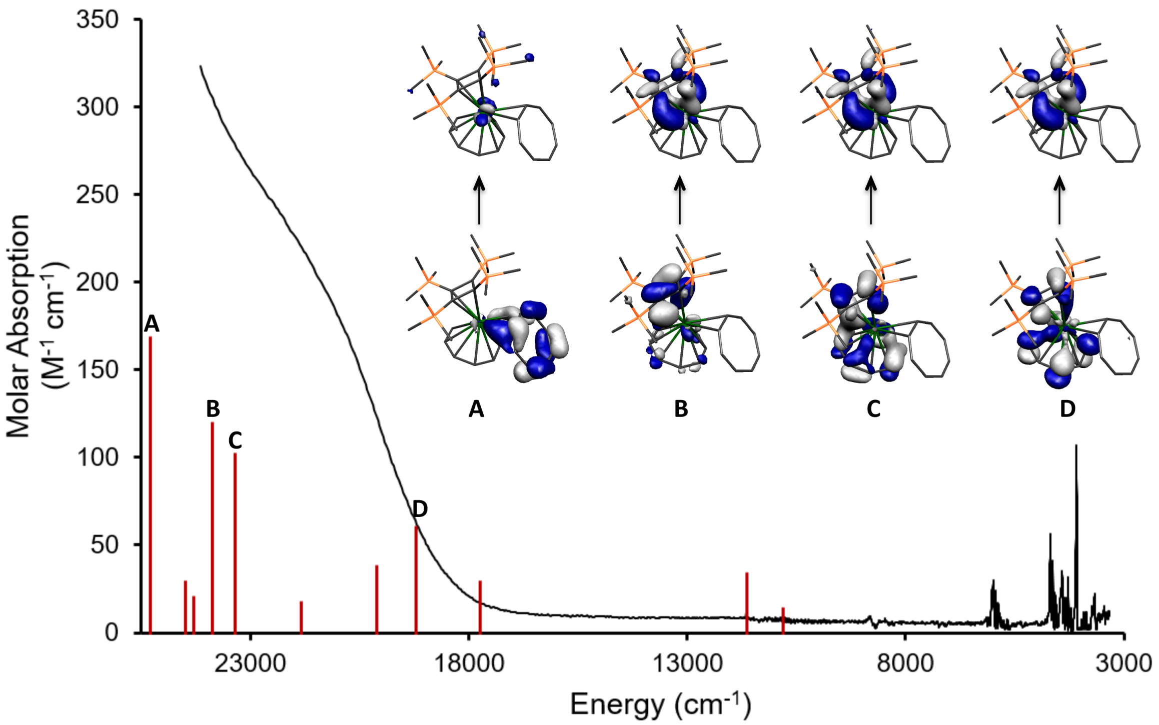

Figure S11. UV/Vis/NIR spectrum of 1 in toluene in the region 3,000-25,000 cm^{-1}. The absorptions in the 3,000-8,000 cm^{-1} region are due to imperfect solvent subtraction. The principal absorptions of interest, in particular A-D, are overlaid with calculated oscillator strengths depicted as vertical red lines. Hydrogen atoms are omitted for clarity. A-D correspond to: A – HOMO−1 to LUMO+8; B – HOMO−5 to LUMO; C – HOMO−4 to LUMO; D – HOMO−3 to LUMO.

Computational Details

General

Restricted geometry optimisations and/or single point energy calculations for **1'** and **1''** were performed using coordinates derived from the crystal structure of **1** as the starting point. No constraints were imposed on the structure during the geometry optimisations. The calculations were performed using the Amsterdam Density Functional (ADF) suite version 2017.[5,6] The DFT geometry optimisations employed Slater type orbital (STO) triple-ζ-plus

polarisation all-electron basis sets (from the Dirac and ZORA/TZP database of the ADF suite). Scalar relativistic approaches were used within the ZORA Hamiltonian for the inclusion of relativistic effects and the local density approximation (LDA) with the correlation potential due to Vosko *et al* was used in all of the calculations.[7] Gradient corrections were performed using the functionals of Becke and Perdew.[8,9] The UV/Vis/NIR spectrum of **1''** was calculated using TD-DFT with the SAOP functional in a benzene continuum. TD-DFT calculations on **1''** reproduce the profile of the observed optical spectrum of **1** very well, but required lowering by a corrective factor of 4,000 cm^{-1} to accommodate the formal 2- charge of **1''**. MOLEKEL[10] was used to prepare the three-dimensional plots of the electron density. The Atoms in Molecules analysis[11,12] was carried out with Xaim-1.0.[13] Since the DFT calculations provide an appropriate description of the electronic structure of the formulations studied CASSCF calculations were not required.

Table S1. Final Coordinates and Energy from a Single Point Energy Calculation on the Geometry Optimised 1'

C	-1.831636	-2.515200	-3.424982
C	1.068187	0.725850	-3.243557
C	-0.137269	1.476032	-3.194163
C	-3.930334	0.315278	-2.792277
C	2.271035	0.708357	-2.488038
C	-0.638686	2.518199	-2.368688
C	0.732007	-3.174300	-2.008022
C	2.763093	1.429049	-1.368403
C	-0.144589	3.238988	-1.248476
C	-1.975698	-4.478366	-1.145855
C	2.261072	2.468008	-0.541379
C	1.057054	3.219211	-0.491826
C	-4.386467	1.921968	-0.300965
C	-1.342502	-1.513280	-0.429897
C	-5.245554	-1.027934	-0.378291
C	-2.255268	-0.391314	-0.157431
C	-0.640967	-1.272366	0.883744
C	-1.594774	-0.128212	1.135221
C	0.884340	-4.031652	1.341070
C	-1.945579	2.822673	2.238957

C	-1.441843	-3.212307	3.137892
C	-3.782664	0.551050	3.245791
C	1.349922	-1.888994	3.314178
C	-0.873141	0.732149	4.079941
H	-1.460982	-3.295468	-4.110066
H	1.042120	-0.054967	-4.005287
H	-1.510845	-1.543375	-3.827687
H	-0.874539	1.128626	-3.919012
H	-2.928916	-2.555641	-3.453011
H	-3.280358	1.123581	-3.160524
H	-3.631907	-0.610319	-3.298967
H	2.951472	-0.083309	-2.806980
H	-4.959585	0.551022	-3.107917
H	1.143195	-2.386543	-2.662044
H	-1.667480	2.784916	-2.613383
H	0.886390	-4.133971	-2.527794
H	3.735016	1.062509	-1.032300
H	-1.751445	-5.304503	-1.840119
H	-3.066853	-4.331517	-1.138611
H	1.332617	-3.201069	-1.089635
H	-0.883584	3.928492	-0.837447
H	-3.669283	2.686903	-0.635030
H	-5.364558	2.175998	-0.740104
H	-5.044483	-2.035318	-0.772749
H	-6.230639	-0.706782	-0.752944
H	2.939168	2.713270	0.278461
H	-1.683921	-4.792158	-0.134538
H	1.026938	3.906150	0.355782
H	0.316312	-4.541604	0.554632
H	-4.485013	2.006385	0.788258
H	-5.304964	-1.111495	0.717029
H	1.872073	-3.769180	0.934002
H	-2.508314	3.127819	1.346500
H	1.055884	-4.762064	2.148465
H	-2.210452	-3.626360	2.466415
H	-0.898103	3.139535	2.102020
H	-4.560058	0.693172	2.482764
H	-2.344341	3.389365	3.096440
H	-1.925246	-2.427054	3.738067
H	-1.116939	-4.013031	3.821797
H	2.256238	-1.578016	2.770637
H	-3.833695	-0.498639	3.572309
H	-4.035387	1.189649	4.107667
H	0.176953	0.930127	3.820418
H	1.641955	-2.717250	3.981180
H	1.032756	-1.054827	3.950351
H	-0.932169	-0.267281	4.530351
H	-1.158786	1.461730	4.854842
Si	-1.125379	-2.872749	-1.698246
Si	-3.885500	0.189632	-0.897708

Si	0.012325	-2.530123	2.119851
Si	-2.042602	0.953089	2.603721
Th	0.201158	0.581242	-0.635958

Energy: –400.70291095 eV

Table S2. Single Point Energy Calculation on 1'

C -1.7727 -2.4821 -3.4273
C 0.7094 0.6067 -3.3043
C -0.5383 1.2377 -3.1322
C -4.0345 0.1943 -2.7059
C 2.0063 0.8529 -2.7965
C -1.0348 2.2963 -2.3363
C 0.7343 -3.1890 -1.9674
C 2.5525 1.7945 -1.8841
C -0.5091 3.1536 -1.3430
C -1.9884 -4.3457 -1.1150
C 2.0507 2.8231 -1.0691
C 0.7818 3.3679 -0.8069
C -4.3843 1.8996 -0.2979
C -1.2669 -1.4422 -0.4749
C -4.9582 -1.0773 -0.1385
C -2.0719 -0.2154 -0.2392
C -0.7230 -1.3461 0.9004
C -1.5280 -0.1337 1.1357
C 0.6903 -4.1500 1.3657
C -1.9000 2.7587 2.0380
C -1.9122 -3.3902 2.7307
C -3.7592 0.5842 3.1640
C 0.7667 -2.2724 3.5880
C -0.9439 0.7865 4.0900
H -1.4698 -3.1873 -4.0368
H 0.6718 -0.1439 -3.8855
H -1.4519 -1.6132 -3.7480
H -1.2108 0.8594 -3.6861
H -2.7522 -2.4768 -3.3951
H -3.6818 1.0242 -3.0897
H -3.5697 -0.5720 -3.1024
H 2.6576 0.2544 -3.1429
H -4.9937 0.1285 -2.8956
H 1.1313 -2.5057 -2.5470
H -1.9504 2.4757 -2.5132
H 0.8259 -4.0687 -2.3894
H 3.4949 1.7069 -1.8054
H -1.6759 -5.1220 -1.6252
H -2.9525 -4.2331 -1.2491
H 1.1952 -3.1919 -1.1025
H -1.1699 3.7097 -0.9476
H -3.8582 2.5975 -0.7412

H -5.3279 1.9958 -0.5436
H -4.7441 -1.9615 -0.5026
H -5.8755 -0.8357 -0.3851
H 2.7365 3.2560 -0.5742
H -1.8052 -4.4861 -0.1625
H 0.7941 4.0234 -0.1194
H 0.2361 -4.4811 0.5631
H -4.2894 1.9866 0.6737
H -4.8772 -1.0976 0.8378
H 1.5930 -3.8499 1.1300
H -2.2719 2.8637 1.1373
H 0.7483 -4.8697 2.0287
H -2.4308 -3.7545 1.9832
H -0.9618 3.0421 2.0389
H -4.3926 0.7015 2.4256
H -2.4086 3.3108 2.6678
H -2.4206 -2.6717 3.1617
H -1.7343 -4.1008 3.3819
H 1.6472 -1.9609 3.2910
H -3.8057 -0.3385 3.4908
H -3.9875 1.2001 3.8914
H -0.0003 0.8818 3.8430
H 0.8742 -3.0662 4.1526
H 0.3257 -1.5638 4.1014
H -1.0883 -0.0935 4.4960
H -1.1858 1.4862 4.7319
Si -1.0901 -2.8058 -1.7050
Si -3.7615 0.1988 -0.8421
Si -0.2829 -2.7067 2.0889
Si -2.0105 0.9492 2.5566
Th 0.4058 0.6090 -0.5291
Energy: –386.32053763 eV

Table S3. Single Point Energy Calculation on 1″

C	-1.77272	-2.48211	-3.42728
C	0.70943	0.60671	-3.30430
C	-0.53827	1.23766	-3.13224
C	-4.03449	0.19427	-2.70592
C	2.00627	0.85286	-2.79649
C	-1.03483	2.29629	-2.33625
C	0.73427	-3.18896	-1.96741
C	2.55248	1.79452	-1.88410
C	-0.50909	3.15359	-1.34303
C	-1.98839	-4.34572	-1.11497
C	2.05067	2.82311	-1.06913
C	0.78184	3.36785	-0.80688
C	4.78796	-0.17058	-0.44281

C	-4.38427	1.89957	-0.29786
C	-1.26691	-1.44224	-0.47490
C	-4.95822	-1.07725	-0.13852
C	3.59494	-0.86327	-0.21388
C	5.36735	1.00353	0.08481
C	-2.07186	-0.21543	-0.23919
C	2.50811	-0.68678	0.67398
C	4.97280	1.99580	0.99243
C	-0.72299	-1.34613	0.90037
C	-1.52805	-0.13369	1.13573
C	0.69026	-4.14996	1.36566
C	3.82397	2.25903	1.76890
C	2.10404	0.32646	1.59460
C	2.61332	1.57954	1.99866
C	-1.89996	2.75874	2.03796
C	-1.91220	-3.39021	2.73072
C	-3.75918	0.58421	3.16402
C	0.76673	-2.27235	3.58802
C	-0.94389	0.78645	4.08999
H	-1.46979	-3.18727	-4.03681
H	0.67181	-0.14389	-3.88551
H	-1.45194	-1.61319	-3.74796
H	-1.21081	0.85941	-3.68612
H	-2.75219	-2.47680	-3.39515
H	-3.68176	1.02421	-3.08967
H	-3.56967	-0.57204	-3.10240
H	2.65761	0.25444	-3.14293
H	-4.99366	0.12845	-2.89560
H	1.13125	-2.50568	-2.54704
H	-1.95040	2.47566	-2.51317
H	0.82591	-4.06875	-2.38943
H	3.49488	1.70691	-1.80536
H	-1.67592	-5.12205	-1.62523
H	-2.95253	-4.23311	-1.24911
H	5.33166	-0.58380	-1.10324
H	1.19519	-3.19187	-1.10252
H	-1.16993	3.70973	-0.94763
H	-3.85815	2.59746	-0.74116
H	3.49143	-1.61949	-0.77958
H	-5.32791	1.99580	-0.54358
H	-4.74406	-1.96150	-0.50263
H	-5.87549	-0.83574	-0.38513
H	2.73654	3.25601	-0.57424
H	6.23887	1.15925	-0.25989
H	-1.80519	-4.48609	-0.16245
H	0.79411	4.02340	-0.11940
H	0.23613	-4.48107	0.56308
H	-4.28937	1.98656	0.67367
H	-4.87717	-1.09755	0.83779
H	1.90128	-1.41742	0.65180

H	5.64567	2.65495	1.11560
H	1.59300	-3.84986	1.12995
H	-2.27188	2.86371	1.13727
H	0.74827	-4.86973	2.02865
H	-2.43083	-3.75445	1.98322
H	-0.96184	3.04208	2.03895
H	3.88111	3.07998	2.24366
H	1.29769	0.10325	2.04476
H	-4.39264	0.70150	2.42564
H	2.00815	2.06991	2.54271
H	-2.40862	3.31085	2.66783
H	-2.42064	-2.67166	3.16168
H	-1.73433	-4.10077	3.38191
H	1.64724	-1.96087	3.29103
H	-3.80568	-0.33851	3.49079
H	-3.98751	1.20008	3.89140
H	-0.00032	0.88177	3.84295
H	0.87416	-3.06620	4.15255
H	0.32570	-1.56380	4.10145
H	-1.08825	-0.09355	4.49603
H	-1.18578	1.48621	4.73191
Si	-1.09009	-2.80578	-1.70504
Si	-3.76149	0.19881	-0.84206
Si	-0.28289	-2.70671	2.08891
Si	-2.01053	0.94915	2.55656
Th	0.40585	0.60905	-0.52909

Energy: –481.98569175 eV

References

1. B. M. Day, F. -S. Gui, S. R. Giblin, A. Sekiguchi, A. Mansikkamäki, R. A. Layfield, *Chem. Eur. J.,* 2018, **24**, 16779.

2. A. Hervé, P. Thueŕy, M. Ephritikhine, J. -C. Berthet, *Organometallics,* 2014, **33**, 2088.

3. T. Cantat, B. L. Scotta and J. L. Kiplinger, *Chem. Commun.,* 2010, **46**, 919.

4. A. Sekiguchi, M. Tanaka, T. Matsuo, H. Watanabe, *Angew. Chem. Int. Ed.,* 2000, **40**, 1675.

5. A. Fonseca Guerra, J. G. Snijders, G. Te Velde, E. J. Baerends, *Theor. Chem. Acc.,* 1998, **99**, 391.

6. G. Te Velde, F. M. Bickelhaupt, S. J. van Gisbergen, A. C. Fonseca Guerra, E. J. Baerends, J. G. Snijders, T. Ziegler, *J. Comput. Chem.,* 2001, **22**, 931.
7. S. H. Vosko, L. Wilk, M. Nusair, *Can. J. Phys.,* 1980, **58**, 1200.
8. A. D. Becke, *Phys. Rev. A.,* 1988, **38**, 3098.
9. J. P. Perdew, *Phys. Rev. B.,* 1986, **33**, 8822.
10. S. Portmann, H. P. Luthi, *Chimia,* 2000, **54**, 766.
11. R. F. W. Bader, *Atoms in Molecules: A Quantum Theory*, Oxford University Press, New York, 1990.
12. R. F. W. Bader, *J. Phys. Chem. A,* 1998, **102**, 7314.
13. http://www.quimica.urv.es/XAIM.
